# Development and validation of a patient-level model to predict dementia across a network of observational databases

**DOI:** 10.1186/s12916-024-03530-9

**Published:** 2024-07-29

**Authors:** Luis H. John, Egill A. Fridgeirsson, Jan A. Kors, Jenna M. Reps, Ross D. Williams, Patrick B. Ryan, Peter R. Rijnbeek

**Affiliations:** 1 Department of Medical Informatics, Erasmus University Medical Center, Rotterdam, The Netherlands; 2 Janssen Research and Development, Raritan, NJ USA

**Keywords:** Dementia prediction, Logistic regression model, External validation, Observational data

## Abstract

**Background:**

A prediction model can be a useful tool to quantify the risk of a patient developing dementia in the next years and take risk-factor-targeted intervention. Numerous dementia prediction models have been developed, but few have been externally validated, likely limiting their clinical uptake. In our previous work, we had limited success in externally validating some of these existing models due to inadequate reporting. As a result, we are compelled to develop and externally validate novel models to predict dementia in the general population across a network of observational databases. We assess regularization methods to obtain parsimonious models that are of lower complexity and easier to implement.

**Methods:**

Logistic regression models were developed across a network of five observational databases with electronic health records (EHRs) and claims data to predict 5-year dementia risk in persons aged 55–84. The regularization methods L1 and Broken Adaptive Ridge (BAR) as well as three candidate predictor sets to optimize prediction performance were assessed. The predictor sets include a baseline set using only age and sex, a full set including all available candidate predictors, and a phenotype set which includes a limited number of clinically relevant predictors.

**Results:**

BAR can be used for variable selection, outperforming L1 when a parsimonious model is desired. Adding candidate predictors for disease diagnosis and drug exposure generally improves the performance of baseline models using only age and sex. While a model trained on German EHR data saw an increase in AUROC from 0.74 to 0.83 with additional predictors, a model trained on US EHR data showed only minimal improvement from 0.79 to 0.81 AUROC. Nevertheless, the latter model developed using BAR regularization on the clinically relevant predictor set was ultimately chosen as best performing model as it demonstrated more consistent external validation performance and improved calibration.

**Conclusions:**

We developed and externally validated patient-level models to predict dementia. Our results show that although dementia prediction is highly driven by demographic age, adding predictors based on condition diagnoses and drug exposures further improves prediction performance. BAR regularization outperforms L1 regularization to yield the most parsimonious yet still well-performing prediction model for dementia.

**Supplementary Information:**

The online version contains supplementary material available at 10.1186/s12916-024-03530-9.

## Background

Dementia is an umbrella term to describe various illnesses that affect cognition and may lead to mental degradation [1]. All types of dementia are progressive, meaning that symptoms may be relatively mild at first but worsen with time, usually over the course of several years. Symptoms include problems with memory, thinking, problem-solving or language, changes in emotion, perception, or behavior. Although getting older is the most significant risk factor for dementia, there exist preventative strategies that may slow down dementia progression. These include physical activity, healthy eating, no smoking or drinking of alcohol, and staying mentally and socially active [2, 3]. Therefore, a prediction model can be a useful tool to quantify the risk of a patient developing dementia in the next years and take risk-factor-targeted intervention [1].

Many patient-level prediction models for identifying individuals who are at risk of dementia have been developed, but only few have been externally validated [4–6]. In our previous work, we highlighted that the lack of validation can largely be attributed to inadequate model reporting, which likely limits clinical uptake of many promising models [7]. Our limited success in achieving satisfactory external validation performance for some of the existing dementia prediction models indicates the need for a more transparent and reproducible approach, leading us to develop a novel model to predict dementia. The Observational Health Data Science and Informatics (OHDSI) initiative has developed extensive infrastructure to facilitate development and validation of patient-level prediction models using observational healthcare data [8, 9]. These include a standardized data structure and vocabularies, and an analytical framework that enforces established best practices for internal and external validation.

In this study, we leverage OHDSI tools to develop and validate logistic regression models to predict dementia in the general population across a network of observational databases. Our objective is to create parsimonious models, achieved through the regularization methods L1 and Broken Adaptive Ridge (BAR). Parsimonious models have the advantage of being easier to implement and therefore are more likely to be clinically useful. Additionally, we assess three candidate predictor sets to optimize prediction performance.

## Methods

### Source of data

This study used observational healthcare data from administrative claims and electronic health records (EHR). These type of data generally do not include dedicated cognition tests, genetic or imaging data, and commonly used variables such as education, which have previously been found to be predictive. However, studies have shown good internal validation performance when developing models on observational data and also found enhanced model applicability in real-world settings [10].

Table 1 presents the five observational healthcare databases that were included in this study. The databases were mapped to the Observational Medical Outcomes Partnership Common Data Model (OMOP CDM) [11]. The OMOP CDM provides a standardized data structure and vocabulary, which enables computer-executed analyses to be shared among researchers and institutions, facilitating external validation of prediction models.

**Table 1:** Data sources that are used for model development and external validation. All data sources have been mapped to the OMOP CDM

Database	Acronym	Person count (in millions)	Country	Data type	Time period
IBM MarketScan® Medicare Supplemental Database (version 2322)	MDCR	10.8	United States	Claims	01/2000–10/2022
Iqvia Disease Analyzer Germany (version 2352)	IQGER	32.1	Germany	GP, EHR	10/2012–09/2022
Optum’s de-identified Clinformatics® Data Mart Database (version 2327)	OPSES	94.8	United States	Claims	05/2000–08/2022
Optum® de-identified Electronic Health Record dataset (version 2247)	OPEHR	107.8	United States	EHR	01/2007–03/2022
Integrated Primary Care Information (version N)	IPCI	2.7	Netherlands	GP	01/2006–12/2022

IBM MarketScan® Medicare Supplemental Database (MDCR) includes data from the health services of retirees in the United States with Medicare supplemental coverage through employer-sponsored plans. The Iqvia Disease Analyzer Germany (IQGER) database consists of mostly primary care data collected from German practices and medical centers for all ages. Optum’s de-identified Clinformatics® Data Mart Database (OPSES) is derived from administrative health claims for members of large commercial and Medicare Advantage health plans in the United States. Optum® de-identified Electronic Health Record dataset (OPEHR) represents longitudinal EHR data derived from dozens of healthcare provider organizations in the United States. The Integrated Primary Care Information (IPCI) database is a Dutch database containing the complete medical record of patients provided by around 350 general practitioners (GP) geographically spread over the Netherlands [12].

### Participants

The target cohort of our study consists of individuals aged 55–84 with an index date between 1 January 2014 and 31 December 2014. This allows for the 5-year follow-up period to end by 31 December 2019, thus avoiding potential irregularities in the data caused by the COVID-19 pandemic. We use the earliest recorded visit to a healthcare provider as the index event.

We exclude persons with prior dementia as defined by our outcome. Moreover, we exclude persons with disease records indicating subtypes of dementia, developmental mental disorder, cognitive impairment, or traumatic brain injury. We also exclude persons with a record of any drug included in the Anatomical Therapeutic Chemical Classification System (ATC) code N06D of anti-dementia drugs.

All exclusion criteria are assessed on the full medical history of a person prior to the index date. The detailed target cohort definition can be found in Additional file 1: Appendix A.

#### Population settings

**Fig. 1:**
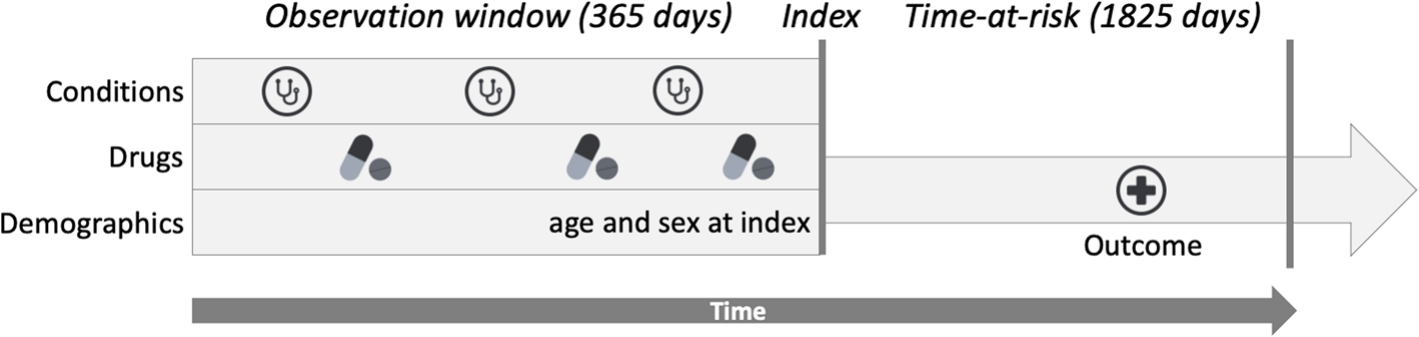
Time windows and index date for the prediction of dementia

Participants require 365 days of continuous observation time before the index date (excluding the index date) in which candidate predictors are assessed (Fig. 1). This relatively short period is consistent with other models in literature that were developed on observational data and enables persons to use the model even if they have not been part of a database for a long time [10, 13]. This limited period of 365 days, as opposed to all-time lookback, was also found to have only small impact on discrimination and calibration as all-time lookback can vary strongly across patients [14].

Moreover, following the recommendations of an empirical analysis of dealing with patients who are lost to follow-up, we allow patients to leave the cohort at any time during the time-at-risk period as long as they have at least 1 day time-at-risk after index [15]. The time-at-risk period for a patient ends after 5 years following the index date.

### Outcome

We investigate the outcome of dementia for the first time in a person’s history within 5 years following the index date. We anticipate this amount of time will mitigate the risk of false negative cases caused by delayed entry of records into the database [16].

Dementia is defined as its concept code in the OMOP CDM and all hierarchical descendants of these concepts according to the SNOMED medical terms hierarchy. Various other concepts that are not direct descendants of the dementia concept are also used to define dementia, such as senility, organic mental disorder, diffuse Lewy body disease, cerebral degeneration associated with another disorder, amnestic disorder, or age-related cognitive decline.

The detailed outcome cohort definition can be found in Additional file 1: Appendix B.

### Statistical analysis methods

We used the OHDSI patient-level prediction framework for model development and validation [8]. This framework enables the development of analysis packages in R that can be shared across data sites mapped to the OMOP CDM.

#### Predictors

This study assesses models with three sets of candidate predictors to predict the health outcome of dementia.

The first set, referred to as the baseline set uses only age groups and sex as candidate predictors. This approach provides a minimalistic model which increases interpretability but may not capture all the relevant information needed for accurate predictions [17].

The second set of predictors, the full set, includes all available candidate predictors from the condition and drug tables in the databases, as well as age groups and sex. This approach aims to capture as much information as possible, which may lead to improved predictive performance. However, the full set may also include irrelevant predictors that can introduce noise and reduce model interpretability, as well as hinder external validation.

To address the issues of both these predictor sets, we also investigate a third set of candidate predictors, the phenotype set, which includes a set of 49 clinically relevant predictors that have been defined in the form of complex phenotypes (described in Additional file 1: Appendix C). These predictors are based on phenotypes defined in the OHDSI phenotype library [18]. We expect that this set may balance prediction performance, interpretability, and ease of validation, while simultaneously reducing noise and redundancy.

Within the parsimonious predictor set, we develop additional models that use interactions between the age group covariate and each of the other covariates. Since age is expected to be among the most predictive covariates for dementia, its interaction with the other covariates allows us to evaluate synergistic effects that may enhance model performance, without the need for additional data collection. The use of covariate interactions also aligns with our parsimony objective, if in a computerized deployment such interactions could be computed automatically to ensure no extra workload for healthcare workers.

The predictors are indicative of whether a patient’s medical history includes a documented diagnosis or prescription, denoted by values of 1 for recorded and 0 for not recorded. It is important to note that instances exist where diagnoses or prescriptions might not always be documented, resulting in a recorded value of 0 to signify the absence of such records. Thus, missing data is treated as though the specific information has not been recorded, without resorting to any form of imputation. Preprocessing of data included removal of covariates with less than 0.1% prevalence in the target cohort and removal of redundant covariates. The latter concerns covariates that have the same value for all persons as well as one-hot-encoded categorical variables such as age or sex.

#### Sample size

Sufficient data availability is a critical prerequisite for reliable prediction [19]. We generate learning curves to determine whether sufficient data is available. We hypothesize that since our target cohort definition is aimed at the general population and does not consider comorbidities as inclusion criteria, large cohort sizes can be obtained from the available data sources (Table 1). Previous work also indicated that sample sizes beyond 5000 persons with the outcome have little impact on further improving model performance. As a result, the decision has been made to limit the sample size to a maximum of 1 million patients, if available, which should ensure a sufficient number of persons with the outcome [19]. Learning curves should approach a plateau if sufficient data is available.

#### Prediction and regularization

The general statistical model of logistic regression has been originally developed and popularized as early as 1944 [20]. Logistic regression remains a state-of-the-art method to develop robust clinical prediction models, despite the impressive advances in more complex prediction approaches such as deep learning [21, 22]. We trained logistic regression models using two types of regularization: L1 regularization and Broken Adaptive Ridge (BAR).

L1 regularization, also referred to as the least absolute shrinkage and selection operator (LASSO), is a widely used method that penalizes the absolute value of the regression coefficients, leading to sparser models with only a subset of predictors having non-zero coefficients. BAR is a novel method that adapts the degree of regularization based on the level of multicollinearity among the candidate predictors, generally resulting in models with very few predictors [23, 24].

In addition, for L1 we employed an adaptive search method to automatically tune the degree of regularization to balance between model complexity and generalization performance on the internal validation set [22]. BAR incorporates the Bayesian information criterion (BIC) to determine its penalty [23].

#### Evaluation

For internal validation, we used a train-test split based on individual persons. Each person appeared only once in the datasets because we only use their earliest visit to a healthcare provider. In each dataset, a random sample of 75% of persons was used to develop the prediction models and the remaining 25% were used to internally validate the models.

To evaluate the performance, we calculated the discrimination of the model using the area under the receiver operating characteristic curve (AUROC) and the model calibration using the E_avg_ metric. The AUROC indicates the probability that for two randomly selected patients, the patient who gets the outcome will be assigned a higher risk. The model calibration is generally presented in a plot to examine agreement between predicted and observed risk across deciles of predicted risk. Calibration assessment is then performed visually which provides a good impression of the direction and scale of miscalibration. Due to the scale of this analysis, we decided to use the single value metric E_avg_ which allows us to compare calibration across models more conveniently. E_avg_ is closely related to Harrell’s E_max_, which is the maximal absolute difference between the smoothed calibration curve and the diagonal line of the best fit [25, 26]. E_avg_ is the average absolute difference between observed and predicted probabilities [25, 26].

To perform external validation, we applied the models to persons matching our target cohort definition in the remaining data sources detailed in Table 1. We examined the external validation performance using AUROC and calibration on the entire external validation data set. In addition, to assess model performance over time, we validate phenotype models on more recent data from patients with an index date in 2015, 2016, and 2017. For the external validation, the models have been recalibrated using weak calibration.

This study was conducted and reported according to the Transparent Reporting of a multivariate prediction model for Individual Prediction or Diagnosis (TRIPOD) guidelines and adhered to the open science principles for publicly prespecifying and tracking changes to study objects, protocol and code as described in the book of OHDSI [27, 28].

## Results

### Participants

Implementing the exclusion and inclusion criteria with a requirement for 365 days of continuous observation time before the index date results in cohort sizes for MDCR, IQGER, OPSES, OPEHR, and IPCI of 1,552,867, 1,486,152, 2,839,676, 7,924,789, and 186,820, respectively. Further sampling one million patients and requiring a minimum of 1 day time-at-risk after the index date results in final participant counts of 999,480, 946,900, 999,439, 971,999, and 186,767, respectively, as detailed in Table 2.

**Table 2:** Characteristics of the patients at baseline across the data sources used for development and external validation

	MDCR	IQGER	OPSES	OPEHR	IPCI
Number of participants	999,480	946,900	999,439	971,999	186,767
Outcomes of dementia (%)	44,800 (4.5)	37,643 (4.0)	47,764 (4.8)	37,978 (3.9)	3094 (1.7)
Median time-at-risk in days (interquartile range)	1043 (1092)	1825 (578)	1748 (1234)	1825 (607)	1825 (443)
Age in years					
55–64 (%)	20,138 (2.0)	360,689 (38.1)	407,419 (40.8)	445,429 (45.8)	81,425 (43.6)
65–74 (%)	643,698 (64.4)	326,407 (34.5)	377,098 (37.7)	299,560 (30.8)	68,150 (36.5)
75–84 (%)	335,644 (33.6)	259,804 (27.4)	214,922 (21.5)	227,010 (23.4)	37,192 (19.9)
Sex					
Male (%)	465,601 (46.6)	412,486 (43.6)	462,293 (46.3)	410,198 (42.2)	86,329 (46.2)
Female (%)	533,879 (53.4)	534,014 (56.4)	537,146 (53.7)	561,801 (57.8)	100,438 (53.8)
Atrial fibrillation (%)	89,837 (9.0)	15,030 (1.6)	67,115 (6.7)	58,983 (6.1)	3708 (2.0)
Any cancer excl. prostate (%)	333,146 (33.3)	74,010 (7.8)	293,895 (29.4)	143,119 (14.7)	22,008 (11.8)
Acute kidney injury (%)	18,035 (1.8)	269 (0.0)	16,398 (1.6)	12,100 (1.2)	0
Kidney disease or end stage renal disease (%)	161,547 (16.2)	37,157 (3.9)	177,023 (17.7)	94,179 (9.7)	295 (0.2)
Heart failure (%)	89,876 (9.0)	24,909 (2.6)	79,433 (8.0)	47,584 (4.9)	3295 (1.8)
Diabetes mellitus type 1 (%)	13,793 (1.4)	3113 (0.3)	10,388 (1.0)	4501 (0.5)	366 (0.2)
Diabetes mellitus type 2 (%)	259,816 (26.0)	89,250 (9.4)	250,890 (25.1)	161,918 (16.7)	22,797 (12.2)
Deep vein thrombosis (%)	10,065 (1.01)	1626 (0.2)	7562 (0.8)	5351 (0.6)	402 (0.2)
Gastrointestinal bleeding (%)	23,846 (2.4)	3653 (0.4)	21,471 (2.2)	12,507 (1.3)	2196 (1.2)
Hyperlipidemia (%)	617,057 (61.7)	83,883 (8.9)	638,339 (63.9)	406,671 (41.8)	17,266 (9.24)
Hypertension (%)	658,451 (65.9)	208,131 (22.0)	602,851 (60.3)	431,709 (44.4)	55,159 (29.5)
Hypothyroidism (%)	169,752 (17.0)	25,907 (2.7)	202,814 (20.3)	113,963 (11.7)	5523 (3.0)
Obesity (%)	96,489 (9.7)	36,225 (3.8)	173,500 (17.4)	349,965 (36.0)	27,593 (14.8)
Osteoporosis (%)	118,901 (11.9)	32,121 (3.4)	129,166 (12.9)	61,805 (6.4)	6079 (3.3)
Pneumonia (%)	32,499 (3.3)	5818 (0.6)	28,113 (2.8)	18,286 (1.9)	4068 (2.2)
Rheumatoid arthritis (%)	27,072 (2.7)	12,114 (1.3)	29,539 (3.0)	17,057 (1.8)	2797 (1.5)
Osteoarthritis (%)	411,328 (41.5)	111,717 (11.8)	402,366 (40.3)	209,548 (21.6)	23,317 (12.5)
Asthma (%)	93,485 (9.4)	23,621 (2.5)	103,129 (10.3)	62,195 (6.4)	10,113 (5.4)

MDCR provides the oldest population with most persons 65 years or older, because the database consists of retirees. IPCI is the only database that provides fewer than one million patient records for the target cohort and is not further sampled. In IQGER, there were 400 persons without a record of biological sex. Acute kidney injury was not commonly found in GP data, evident from the outcome rates in IQGER and IPCI. With a median time-at-risk of 1043 days, MDCR database has shorter continuous observation time than the other databases, possibly due to the older population. The outcome rate of dementia is consistent across all databases, except IPCI for which it is considerably lower.

### Sample size

We generated learning curves with subsets that included up to 5000 persons with the outcome. The aim was not to determine an exact sample size but to assess whether sufficient data is available. Learning curves were generated using L1 regularization for the full set of candidate predictors including age, sex, condition occurrences, and drug exposures.

**Fig. 2:**
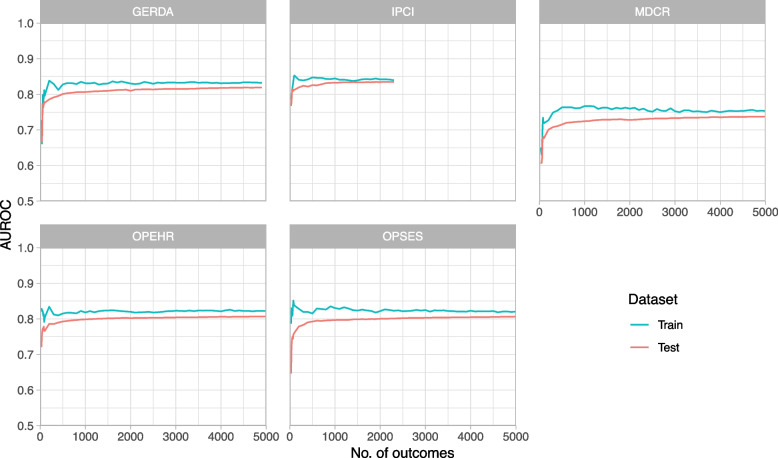
Learning curves using the full set and L1 regularization for up to 5000 persons with the outcome

Figure 2 shows that learning curves are in the plateau phase even for the IPCI database, which provides the smallest dataset with just over 2300 persons with the outcome in its training set. In the context of L1 regularization on the full set of candidate predictors, learning curves suggest no substantial overfitting across databases, as shown by the similar performances on training and test sets.

### Internal and external prediction performance

#### Discrimination performance

**Fig. 3:**
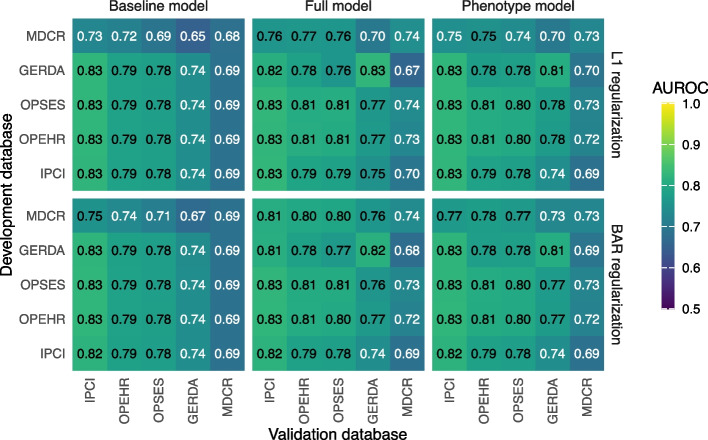
Internal and external discrimination performance (AUROC) of baseline models, full models, and phenotype models for L1 and BAR regularization

Figure 3 illustrates the internal and external discrimination performance of baseline models, full models, and phenotype models for L1 and BAR regularization methods. Generally, models perform best on their development databases, with external validation performance decreasing. Internal discrimination performance indicates that the baseline models perform best on IPCI data and worst on MDCR, with negligible influence from the regularization method. Full models also perform best on IPCI data and worst on MDCR data. The MDCR model trained with L1 performs worst across the other databases, while the MDCR model with BAR sees improvements in transportability. Internal and external discrimination performance of the phenotype models is consistent with the full models. The phenotype models using covariate interactions demonstrate similar discrimination performance to the original phenotype models, as detailed in Additional file 1: Appendix D. Moreover, model performance of the phenotype models was assessed on more recent data from patients with index date in 2015, 2016, and 2017. Discrimination performance remained stable, whereas calibration performance showed a slight decline over time, as detailed in Additional file 1: Appendix E.

#### Calibration performance

**Fig. 4:**
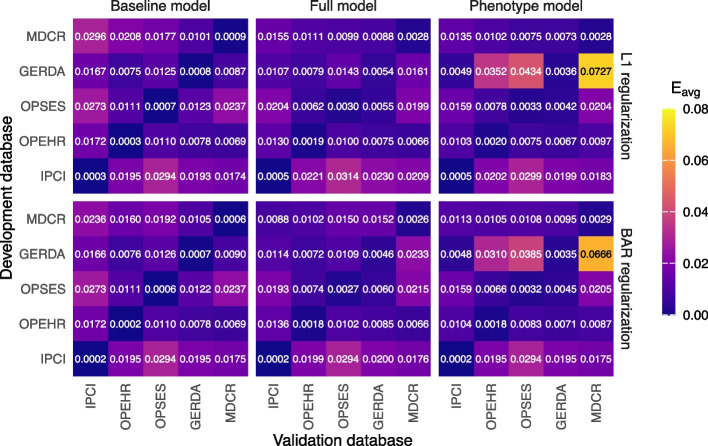
Internal and external calibration performance (E_avg_) of baseline models, full models, and phenotype models for L1 and BAR regularization

Figure 4 illustrates the internal and external calibration performance of baseline models, full models, and phenotype models for L1 and BAR regularization methods. Looking at E_avg_ performance, models are calibrated best on their development databases, with external validation performance decreasing. This is despite the effort to recalibrate models for external databases using the weak calibration method. The IPCI model appears to be the most poorly calibrated model across the external data sources. Calibration of the MDCR models in external data sources is better than for the baseline model trained on MDCR. One exception to this is the IQGER model for the phenotype predictor set which performs worst on external databases. The phenotype models using covariate interactions demonstrate similar calibration performance to the original phenotype models, as detailed in Additional file 1: Appendix D.

### Regularization and predictors

**Fig. 5:**
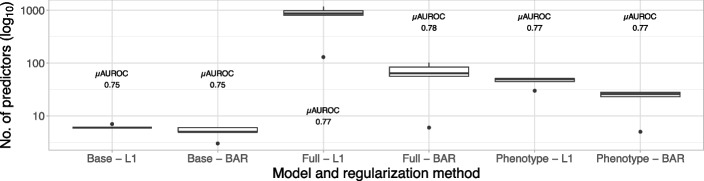
Number of predictors across databases for each model and regularization method. Each boxplot is annotated with the mean discrimination performance (µAUROC), internal and external, across all respective databases

Inspecting the number of predictors (Fig. 5) in the full models, we can observe that the BAR model selected for IQGER, MDCR, OPSES, OPEHR, and IPCI a total of 102, 84, 64, 56, and 6 predictors, respectively. This is considerably less than using L1 regularization with 808, 1172, 987, 877, and 130 predictors on the same respective databases. In total, there were 930, 2416, 2307, 2012, and 1363 candidate predictors available before regularization, respectively.

A similar trend is observed for the phenotype models, where BAR models based on IQGER, MDCR, OPSES, OPEHR, and IPCI contained 28, 26, 28, 23, and 5 predictors, respectively. This is less than using L1 regularization with 45, 51, 52, 50, and 30 predictors on the same respective databases. Phenotype models that incorporate covariate interactions selected 29, 34, 41, 30, and 7 predictors for BAR and 207, 166, 192, 146, and 43 predictors for L1, respectively.

Despite the discrepancy in number of predictors, the phenotype models achieve similar performance as the full models (Fig. 5). The number of predictors for all models can be found in Additional file 1: Appendix F.

## Discussion

Treatment and management of dementia is focused on slowing its progression and improving symptoms. A patient-level model that can reliably predict dementia can support healthcare providers to take risk-factor-targeted interventions at an early stage, potentially improving the quality of life of affected individuals. Identifying optimal model design choices for the candidate predictor set, regularization method, and development data source to improve prediction performance, can ultimately contribute to a more proactive approach to dementia management.

### Participants and data

We generated learning curves to assess sample size requirements. Learning curves reached a plateau across all databases, which suggests that adding more data will likely have minimal impact on improving discrimination performance. We conclude that model development will not suffer from insufficient data and is feasible on our data sets. An argument could be made to sample smaller datasets. However, given that training times and resource requirements for training logistic regression models were found to not be computationally prohibitive, all available data was used [19].

### Internal and external validation

Age is the strongest known risk factor for dementia, as reflected in the discrimination performance of the baseline models shown in Fig. 3. Even without the additional candidate predictors of condition occurrences and drug exposures, the baseline models perform well.

Two observations can be made about the MDCR baseline model: [1] the model performs much worse across the external data sources, and [2] all other models perform poorly on MDCR data. The reason for this is likely the demographic case mix of the data. MDCR is a Medicaid database with an older population compared to the other databases. This is evident in Table 2, where persons aged 55–65 make only up about 2% of its population. As a result, the age range that the MDCR model is trained on narrows, making persons less separable using age as predictor and resulting in poorer discrimination performance. The columns with constant performance in the heat maps (Fig. 3) indicate the same validation performance regardless of the model. We believe this highlights a limitation of using age as sole predictor, as separability of persons for a specific outcome can depend on a database’s case mix. IQGER data seem to suffer from this same phenomenon, but to a smaller degree.

For the full models, the addition of candidate predictors of drugs and conditions can provide improved model performances, both internally and externally. Internal prediction performance improves for models that performed the worst using only age group and sex (MDCR and IQGER), improves slightly for OPEHR and OPSES, and no change is observed for IPCI.

Lastly, the phenotype models perform comparably to the full models. This is a valuable observation, because the phenotype models can have at most 57 predictors (including age groups and sex) but have shown to have even fewer when using regularization (Fig. 5). Incorporating covariate-age interactions does not further improve the discrimination and calibration performance of the phenotype models, nor does it reduce model complexity. Therefore, the models based on the original phenotype set are preferred. Calibration is an important performance metric, which should not be neglected. The E_avg_ is low for all models developed, which indicates good calibration, even across external databases. The heightened E_avg_ performance of the IPCI model in external data can be attributed to lower prevalence of dementia in IPCI data (Table 2). When applied to datasets with higher dementia rates, such models generally underpredict the outcome. Model performance remained stable for the phenotype models when validating on newer data (Additional file 1: Appendix E).

### Regularization and predictors

Prediction models trained on high-dimensional observational data can include a large number of predictors [19]. While many predictors may optimize performance, it can be a barrier to clinical implementation. The utility of models for dementia prediction requires that they can be widely implemented in worldwide healthcare settings. Therefore, we investigated approaches with fewer candidate predictors in the form of the models trained on the base set and phenotype set. Moreover, we investigated regularization methods that perform feature selection such as L1 and BAR.

For the base models, no performance difference is observed between the two regularization methods (L1, BAR). Interestingly, the full models also perform similarly regardless of the type of regularization. For MDCR, external validation performance of the BAR model even improves over models regularized using L1. From Fig. 5, it becomes evident that BAR models are more parsimonious than L1 models, making them the first choice given similar performance, as fewer predictors can improve applicability in clinical practice.

### Which is the best model?

The OPSES and OPEHR models slightly outperform the models from the other databases looking at the average internal and external discrimination performance (Additional file 1: Appendix G). Additionally, the full and phenotype models show equal performance. We can determine the best model by considering predictor count, where fewer is better. This makes the BAR models the most compelling candidates. The OPEHR phenotype model using BAR has the fewest predictors with 24, as compared to the OPEHR full model using BAR with 57 predictors, the OPSES full model using BAR with 65 predictors, and the OPSES phenotype model using BAR with 29 predictors. Moreover, even though calibration is good for all models, the OPEHR models outperform the OPSES models slightly on E_avg_. OPEHR also provides more continuous observation time for patients as evident from the median time-at-risk of 1825 days as compared to OPSES with 1748 days.

Therefore, the OPEHR phenotype model trained using BAR is our most suitable model for dementia prediction and presented in Additional file 1: Appendix H. Although this model cannot be directly compared to existing dementia prediction models due to different cohort definitions and modeling parameters (time-at-risk window, observation window, etc.), we can still assess it in the context of the existing literature. We previously investigated reporting of 59 existing dementia prediction models that were presented in 35 publications [7].

Well-reported models could be fully replicated and applied based on the statistical analysis information reported in the research paper. These include a model by Walters et al. which achieved c-statistic of 0.84 in persons aged 60–79 [10]. However, external validation proved difficult as predictors such as social deprivation or BMI measurements are generally not available in observational data [7]. As a result, external validation performance deteriorates. On MDCR, OPSES, OPEHR, IQGER, and IPCI, we observed AUROC performances of 0.69, 0.74, 0.73, 0.75, and 0.76, respectively, for this model [7]. The OPEHR phenotype model, although getting outperformed on its development data, uses more commonly available predictors in observational data and discriminated better when evaluated on these same databases. Similarly, Nori et al. use L1 regularized logistic regression to train a model on OptumLabs Data Warehouse data which achieves 0.69 AUROC [29]. External validation showed that this model does not transport well to MDCR, OPSES, OPEHR, IQGER, and IPCI, with AUROCs of 0.66, 0.67, 0.62, 0.67, and 0.64, respectively, considerably less than the performance of our phenotype model.

While many of the remaining models that were assessed achieve comparable AUROC to our model, we believe the lack of an external validation makes many of these models less suitable for clinical practice. The final model is presented in Additional file 1: Appendix H.

### Clinical utility

A prediction model has clinical utility when it can aid healthcare professionals in their decision-making and patient management, ultimately resulting in improved patient outcomes. This study addresses several factors to improve clinical utility of our model that include improvement of performance metrics, clinical relevance of predictors derived from routinely collected data, short 1-year continuous observation time, and an external validation.

We avoid the use of qualitative descriptors of model performance for AUROC thresholds as these could be arbitrarily based on digit preference, and therefore the general recommendation is to present AUROC values without labels [30, 31]. However, we acknowledge there is room to further improve discrimination which likely will result in improved clinical utility [10].

Moreover, while we have made considerable strides in developing and validating predictive models, a significant challenge remains in translating these models into practical clinical applications. Currently, despite the availability of various informative models, their implementation into clinical practice has been underwhelming.

Moving forward, it will be crucial to prioritize efforts towards implementing these models into everyday clinical practice, allowing the insights generated to effectively inform and improve dementia management. Thus, our research does not simply end in model development and validation, but prompts further action to ensure our results translate into tangible healthcare improvements.

### Limitations and future work

The benefits and challenges of using observational data for research are well documented. Hersh et al. highlighted the real-world nature and quantity, while acknowledging its potential limitations such as incompleteness, inaccuracies, or insufficient granularity [32]. While we hypothesize that using observational data can enhance clinical utility of a model, it notably excludes established prediction approaches for dementia, such as using brain MRI, cognitive assessment, or plasma Alzheimer biomarkers [33, 34]. Despite the well-established nature of logistic regression for clinical prediction, but considering these different types of data and a seeming performance ceiling we observed for our prediction task, we recognize the need to explore alternative modeling techniques for dementia prediction in the future [21].

Logistic regression may also be constrained by the competing risk of death, an inherent challenge in longitudinal studies focusing on age-related diseases. One precaution we are taking is that our analysis includes those patients that are lost to follow-up, for example, due to death. This approach was found to maintain a comparable performance but avoids bias to the model [15]. However, alternative modeling techniques for dementia prediction that take into account competing risks, such as the Fine-Gray subdistribution hazard model, or models that use time-varying covariates, present a promising direction for future work [35]. Moreover, recent work in deep learning has introduced revised architectures for tabular data, potentially providing a way to identify complex patterns not seen by conventional modeling approaches [36, 37, 38].

## Conclusion

In this study, we developed and externally validated patient-level models to predict dementia. We focused on identifying optimal model design choices for candidate predictor sets, regularization methods, and development data source to improve prediction performance, which can ultimately contribute to a more proactive approach to dementia management. Although demographic age is found to be a key driver for dementia prediction, we demonstrate that additional predictors based on condition diagnoses and drug exposures can further improve prediction performance to varying degrees.

During model development, BAR regularization outperformed L1 regularization to yield the most parsimonious yet still well-performing prediction models. We choose a final model trained on EHR databases which demonstrates good external validation performance across four other observational databases, outperforming previously validated models on the same data.

The low complexity of the chosen model emphasizes its suitability for broader application, holding promise to notably contribute to our understanding and management of dementia in a healthcare setting.

However, despite having made considerable strides in developing and validating predictive models, a significant challenge remains in translating these models into clinical practice.

### Supplementary Information

Additional file 1: Appendix A – Target cohort definition. Appendix B – Outcome cohort definition. Appendix C – Candidate predictor of phenotypes model. Appendix D – Age-covariate interactions. Appendix E – Performance stability over time. Appendix F – Number of predictors. Appendix G – Average internal and external prediction performance. Appendix H – How to calculate the risk of dementia for a new patient

## Data Availability

The Optum and IBM MDCR data that support the findings of this study are available from IBM MarketScan Research Databases (contact at: http://www.ibm.com/us-en/marketplace/marketscan-research-databases) and Optum (contact at: http://www.optum.com/solutions/data-analytics/data/real-world-data-analytics-a-cpl/claims-data.html) but restrictions apply to the availability of these data, which were used under license for the current study, and so are not publicly available. Due to ethical concerns, supporting data cannot be made openly available for IPCI and Iqvia Germany datasets.

## Abbreviations

OHDSI: Observational Health Data Science and Informatics
OMOP CDM: Observational Medical Outcomes Partnership Common Data Model
EHR: Electronic health record
L1: Least absolute shrinkage and selection operator (LASSO)
BAR: Broken Adaptive Ridge
MDCR: IBM MarketScan® Medicare Supplemental Database
IQGER: Iqvia Disease Analyzer Germany
OPSES: Optum’s de-identified Clinformatics® Data Mart Database
OPEHR: Optum® de-identified Electronic Health Record dataset
IPCI: Integrated Primary Care Information
SNOMED: Systematized Nomenclature of Medicine
ATC: Anatomical Therapeutic Chemical Classification System
TRIPOD: Transparent Reporting of a multivariate prediction model for Individual Prediction or Diagnosis
AUROC: Area under the receiver operating characteristic curve
E_avg_: Average absolute difference between observed and predicted probabilities in calibration
E_max_: Maximum absolute difference between observed and predicted probabilities in calibration
