# Supplementary material for: Development and validation of a patient-level model to predict dementia across a network of observational databases
Source: BMC Med. 2024 Jul 29;22:308. doi: 10.1186/s12916-024-03530-9 (PMC11288076; doi:10.1186/s12916-024-03530-9)
Supplement: Supplementary file 1 — Additional file 1: Appendix A – Target cohort definition. Appendix B – Outcome cohort definition. Appendix C – Candidate predictor of phenotypes model. Appendix D – Age-covariate interactions. Appendix E – Performance stability over time. Appendix F – Number of predictors. Appendix G – Average internal and external prediction performance. Appendix H – How to calculate the risk of dementia for a new patient [file 12916_2024_3530_MOESM1_ESM.docx]

# Additional file 1

# Appendix A – Target cohort definition

For the target cohort we included persons between 55-84 years of age with an index between 1 January 2014 – 31 December 2014. We use the earliest recorded visit to a healthcare provider as the index event.

For concept codes as used in the OMOP CDM, we exclude persons with prior dementia as defined by our outcome. Moreover, we exclude patients with disease records indicating subtypes of dementia including Parkinsonism (concept 4140090), Korsakoff’s psychosis (concept 4288013), Huntington’s chorea (concept 374341), human immunodeficiency virus infection (concept 439727), Creutzfeldt-Jakob disease (concept 372241), and all hierarchical descendants of these concepts according to the SNOMED medical terms hierarchy. In addition, we exclude patients with records of developmental mental disorder (concept 4043545), demyelinating disease of central nervous system (concept 375801), and degenerative disease of the central nervous system (concept 4213310).

We exclude persons with a record of any drug included in the ATC code N06D of anti-dementia drugs (concept 21604792).

We exclude persons with a record of traumatic brain injury (concept 4132546), traumatic AND/OR non-traumatic brain injury (concept 4133611), and lesion of brain (concept 4200516), and all hierarchical descendants of these concepts according to the SNOMED medical terms hierarchy.

We exclude persons with cognitive impairment including memory impairment (concept 4304008), impaired cognition (concept 443432), and mild cognitive disorder (concept 4297400), and all hierarchical descendants of these concepts according to the SNOMED medical terms hierarchy.

All exclusion criteria are assessed all time prior to the index date. The full computer-executable definition for the OMOP CDM is available in the *cohorts/target.json* at <https://github.com/mi-erasmusmc/DementiaPredictionModel>

# Appendix B – Outcome cohort definition

We investigate the outcome of dementia for the first time in a person’s history within 5 years following the index date.

For concept codes as used in the OMOP CDM, dementia is defined as senile degeneration of brain (concept 373179), or frontotemporal dementia (concept 4043378), or dementia (concept 4182210), and all hierarchical descendants of these concepts according to the SNOMED medical terms hierarchy.

Moreover, dementia is defined as senility (concept 435088), or organic mental disorder (concept 374009), or diffuse Lewy body disease (concept 380701), or cerebral degeneration associated with another disorder (concept 4104700), or amnestic disorder (concept 372608), or age-related cognitive decline (4009705).

We explicitly exclude from the definition of dementia senile and presenile organic psychotic conditions (concept 4152048), postconcussion syndrome (concept 372610), general paresis – neurosyphilis (concept 377788), drug-induced dementia (concept 376095), dementia following injury caused by exposure to ionizing radiation (concept 42535731), dementia caused by volatile inhalant (concept 37311999), dementia caused by toxin (concept 36717598), or dementia caused by heavy metal exposure (concept 37116464), and all hierarchical descendants of these concepts according to the SNOMED medical terms hierarchy.

The full computer-executable definition for the OMOP CDM is available in the *cohorts/outcome.json* at <https://github.com/mi-erasmusmc/DementiaPredictionModel>

# Appendix C – Candidate predictor of phenotypes model

The full computer-executable definition of the phenotype predictor set (Table C1) for the OMOP CDM is available in the *cohorts/* directory at <https://github.com/mi-erasmusmc/DementiaPredictionModel>.

Table C1. Candidate predictor of phenotype models.

| **Phenotype model candidate predictors** |
| --- |
| acetaminophen exposures |
| alcoholism |
| anemia |
| angina |
| antiepileptics |
| anxiety |
| osteoarthritis |
| aspirin exposures |
| asthma |
| atrial fibrillation |
| hormonal contraceptive exposure |
| cancer |
| acute kidney injury |
| chronic kidney disease |
| heart failure |
| chronic obstructive pulmonary disease |
| coronary artery disease |
| major depressive disorder |
| diabetes mellitus type 1 |
| diabetes mellitus type 2 |
| deep vein thrombosis |
| dyspnea |
| edema |
| gastroesophageal reflux disease |
| acute gastrointestinal bleeding |
| heart valve disorder |
| earliest chronic hepatitis |
| hyperlipidemia |
| hypertension |
| hypothyroidism |
| inflammatory bowel disease |
| lower back pain |
| neuropathy |
| obesity |
| opioid exposure |
| osteoporosis |
| peripheral vascular disease |
| pneumonia |
| acute respiratory failure |
| rheumatoid arthritis |
| seizure |
| sepsis |
| skin ulcer |
| sleep apnea |
| smoking |
| steroid exposure |
| hemorrhagic stroke |
| non-hemorrhagic stroke |
| urinary tract infectious disease |

# Appendix D – Age-covariate interactions

The phenotype models that incorporate age-covariate interactions demonstrate similar discrimination (Figure D1) and calibration (Figure D2) performance to the original phenotype models. Phenotype models developed on IQGER, MDCR, OPSES, OPEHR, and IPCI selected 29, 34, 41, 30, 7 predictors for BAR, and 207, 166, 192, 146, 43 predictors for L1, respectively.

| 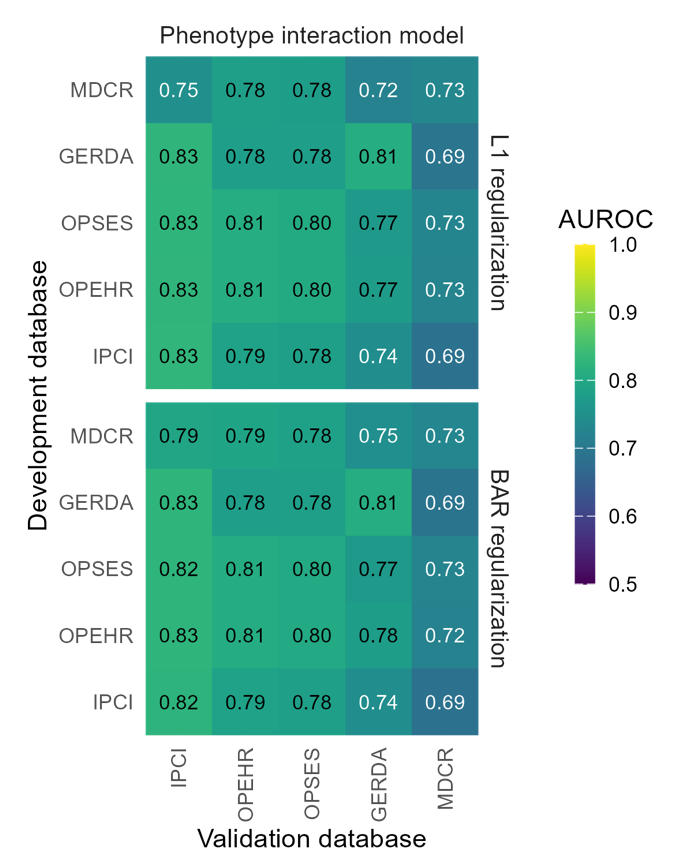  Figure D1. Internal and external discrimination performance (AUROC) of phenotype interaction model for L1 and BAR regularization. | 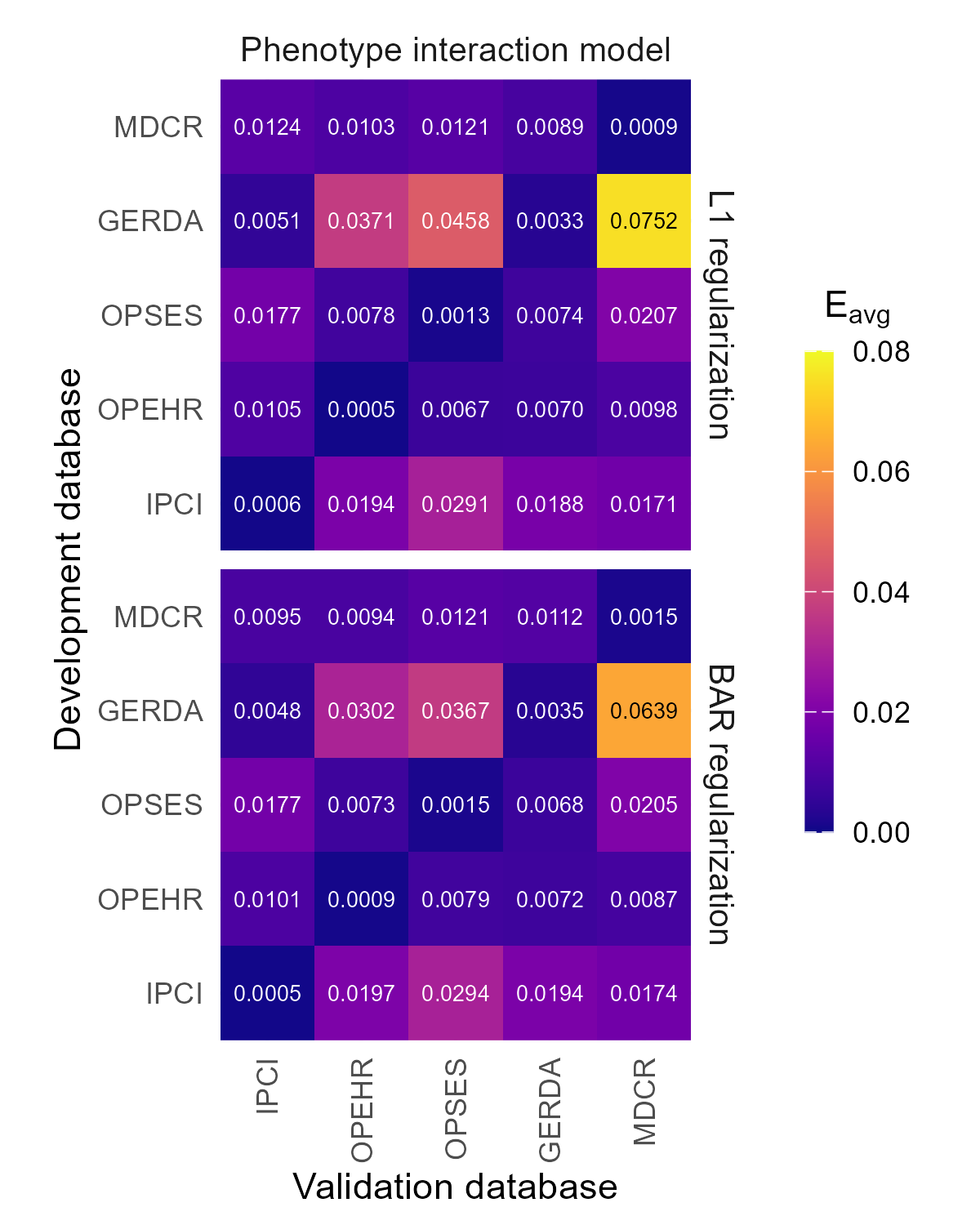  Figure D2. Internal and external calibration performance (E_avg_) of phenotype interaction model for L1 and BAR regularization. |
| --- | --- |

# Appendix E – Performance stability over time

Phenotype models are developed on data from patients with an index date in 2014. To assess performance stability over time, we validate these models on data from patients with an index in 2015, 2016, and 2017. Figure E1 and Figure E2 summarize the discrimination and calibration performance of models developed using BAR and L1 regularization, respectively.


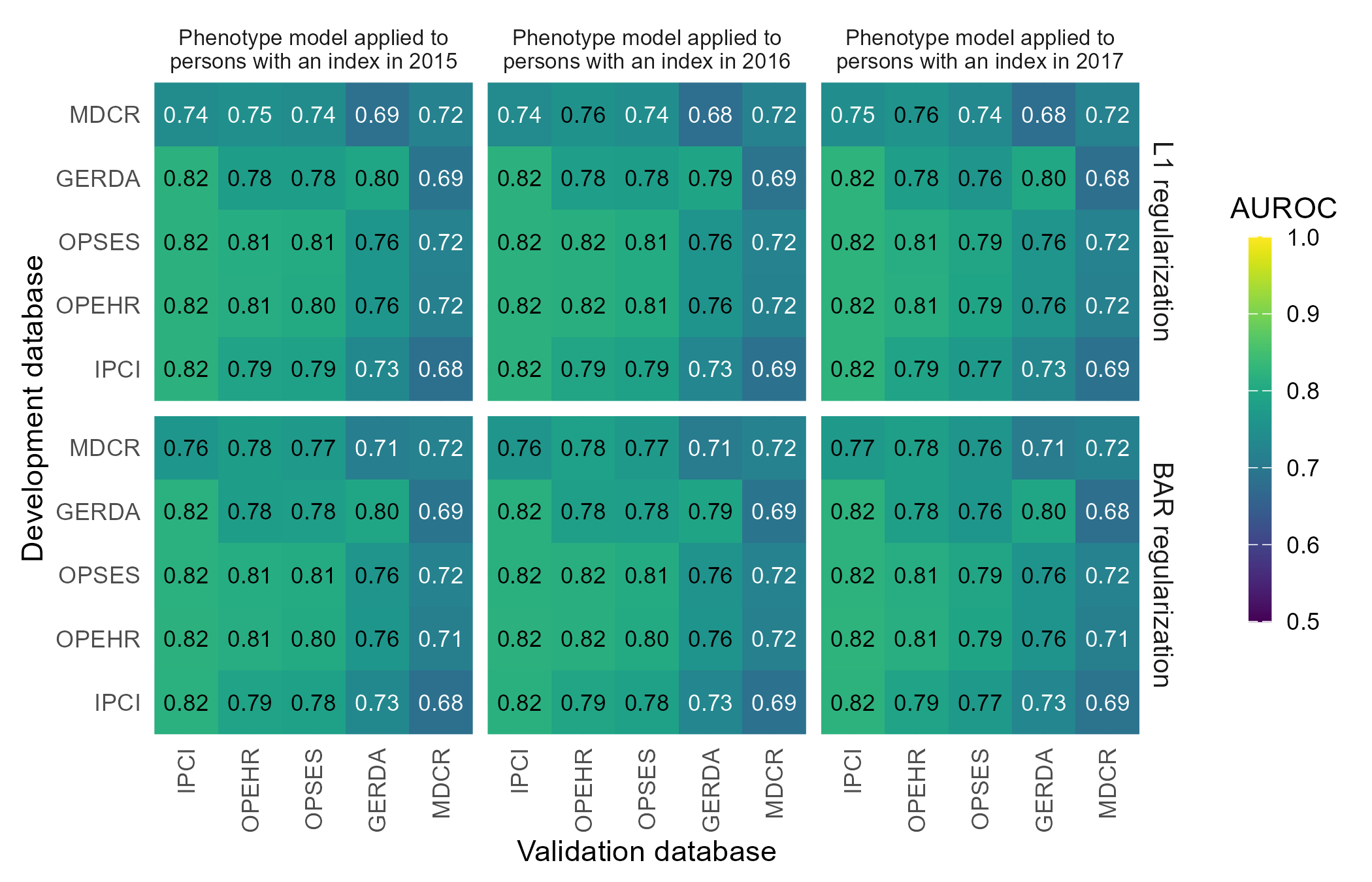


Figure E1. Discrimination performance (AUROC) of phenotype models that were developed on patients with an index date in 2014, on data from 2015, 2016, and 2017.


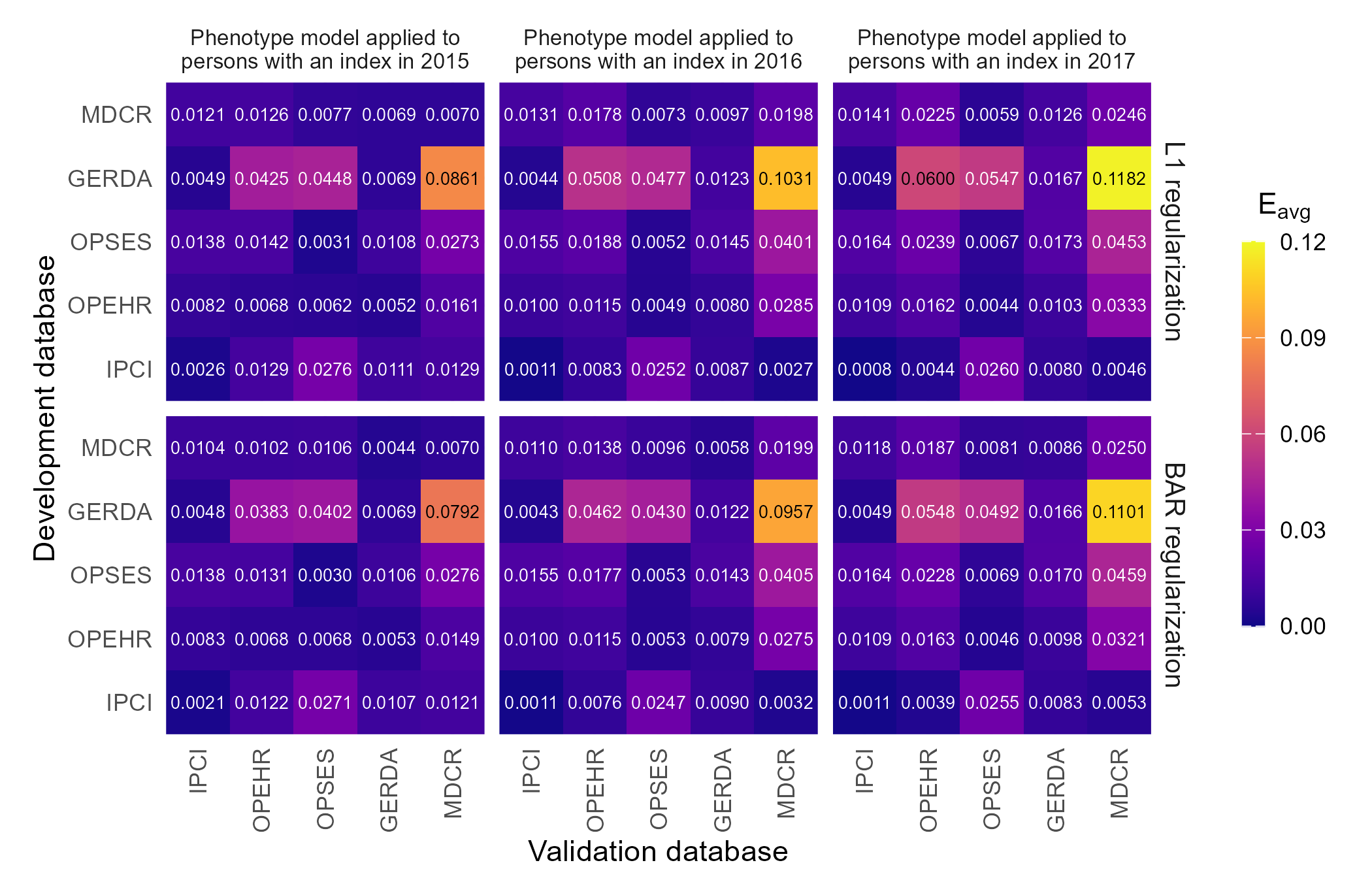


Figure E2. Calibration performance (E_avg_) of phenotype models that were developed on patients with an index date in 2014, on data from 2015, 2016, and 2017.

# Appendix F – Number of predictors

Table F1. Number of model predictors across database, regularization method, and predictor set.

| **Database** | **Model** | **Regularization** | **No. of all covariates (before pre-processing)** | **No. of candidate predictors (after pre-processing)** | **No. of predictors** |
| --- | --- | --- | --- | --- | --- |
| IQGER | Base | L1 | 8 | 7 | 7 |
|  |  | BAR | 8 | 7 | 5 |
|  | Full | L1 | 51392 | 930 | 808 |
|  |  | BAR | 51392 | 930 | 102 |
|  | Phenotype | L1 | 57 | 50 | 45 |
|  |  | BAR | 57 | 50 | 28 |
| MDCR | Base | L1 | 8 | 6 | 6 |
|  |  | BAR | 8 | 3 | 3 |
|  | Full | L1 | 15341 | 2416 | 1172 |
|  |  | BAR | 15341 | 2416 | 84 |
|  | Phenotype | L1 | 57 | 53 | 51 |
|  |  | BAR | 57 | 53 | 26 |
| OPSES | Base | L1 | 8 | 6 | 6 |
|  |  | BAR | 8 | 6 | 6 |
|  | Full | L1 | 15950 | 2307 | 987 |
|  |  | BAR | 15950 | 2307 | 64 |
|  | Phenotype | L1 | 57 | 53 | 52 |
|  |  | BAR | 57 | 53 | 28 |
| OPEHR | Base | L1 | 8 | 6 | 6 |
|  |  | BAR | 8 | 6 | 6 |
|  | Full | L1 | 26982 | 2012 | 877 |
|  |  | BAR | 26982 | 2012 | 56 |
|  | Phenotype | L1 | 57 | 53 | 50 |
|  |  | BAR | 57 | 53 | 23 |
| IPCI | Base | L1 | 8 | 6 | 6 |
|  |  | BAR | 8 | 6 | 5 |
|  | Full | L1 | 6721 | 1363 | 130 |
|  |  | BAR | 6721 | 1363 | 6 |
|  | Phenotype | L1 | 57 | 48 | 30 |
|  |  | BAR | 57 | 48 | 5 |

# Appendix G – Average internal and external prediction performance

Table G1. Average internal and external prediction performance across databases, regularization method, and predictor set.

|  | **Avg. Base L1** | **Avg. Base BAR** | **Avg. Full L1** | **Avg Full BAR** | **Avg. Phenotype L1** | **Avg. Phenotype BAR** |
| --- | --- | --- | --- | --- | --- | --- |
| MDCR | 0.70 | 0.71 | 0.75 | 0.78 | 0.73 | 0.75 |
| IQGER | 0.76 | 0.76 | 0.77 | 0.77 | 0.78 | 0.78 |
| OPSES | 0.76 | 0.76 | 0.79 | 0.79 | 0.79 | 0.79 |
| OPEHR | 0.77 | 0.77 | 0.79 | 0.79 | 0.79 | 0.79 |
| IPCI | 0.77 | 0.76 | 0.77 | 0.76 | 0.77 | 0.76 |

# Appendix H – How to calculate the risk of dementia for a new patient

Table H1. Final phenotype model trained using BAR.

| **Predictor** | **Formula symbol** | **Evaluate for patient as follows** | **Coefficient** |
| --- | --- | --- | --- |
| (Intercept) |  | Takes constant value of -2.8289 | -2.8289 |
| Age group: 55 – 59 | I(a1) | Takes value 1 if patient belongs to age group 55 – 59; takes value 0 otherwise | -2.3499 |
| Age group: 60 – 64 | I(a2) | Takes value 1 if patient belongs to age group 60 – 64; takes value 0 otherwise | -1.8485 |
| Age group: 65 – 69 | I(a3) | Takes value 1 if patient belongs to age group 65 – 69; takes value 0 otherwise | -1.2727 |
| Age group: 70 – 74 | I(a4) | Takes value 1 if patient belongs to age group 70 – 74; takes value 0 otherwise | -0.6051 |
| Age group: 75 – 79 | I(a5) | Takes value 1 if patient belongs to age group 75 – 79; takes value 0 otherwise | 0 |
| Age group: 80 – 84 | I(a6) | Takes value 1 if patient belongs to age group 80 – 84; takes value 0 otherwise | 0.8051 |
| Sex: male | I(male) | Takes value 1 if male; takes value 0 if female | -0.0406 |
| Acetaminophen exposures | I(ace) | Takes value 1 if use of acetaminophen = yes; takes value 0 if use of acetaminophen = no | 0.1630 |
| Seizure | I(sei) | Takes value 1 if patient had a diagnosis of seizure in the past 365 days; takes value 0 if no diagnosis | 0.9237 |
| Anemia | I(ane) | Takes value 1 if patient had a diagnosis of anemia in the past 365 days; takes value 0 if no diagnosis | 0.1856 |
| Antiepileptics exposure | I(epi) | Takes value 1 if use of antiepileptics = yes; takes value 0 if use of antiepileptics = no | 0.2690 |
| Cancer | I(can) | Takes value 1 if patient had a diagnosis of cancer in the past 365 days; takes value 0 if no diagnosis | -0.2363 |
| Major depressive disorder | I(dep) | Takes value 1 if patient had a diagnosis of major depressive disorder in the past 365 days; takes value 0 if no diagnosis | 0.4278 |
| Chronic obstructive pulmonary disease | I(copd) | Takes value 1 if patient had a diagnosis of chronic obstructive pulmonary disease in the past 365 days; takes value 0 if no diagnosis | 0.0766 |
| Heart failure | I(hf) | Takes value 1 if patient had a diagnosis of heart failure in the past 365 days; takes value 0 if no diagnosis | 0.0909 |
| Chronic kidney disease | I(kid) | Takes value 1 if patient had a diagnosis of chronic kidney disease in the past 365 days; takes value 0 if no diagnosis | 0.1216 |
| Hyperlipidemia | I(hyl) | Takes value 1 if patient had a diagnosis of hyperlipidemia in the past 365 days; takes value 0 if no diagnosis | -0.0464 |
| Hypertension | I(hyp) | Takes value 1 if patient had a diagnosis of hypertension in the past 365 days; takes value 0 if no diagnosis | 0.1381 |
| Obesity | I(obe) | Takes value 1 if patient had a diagnosis of obesity in the past 365 days; takes value 0 if no diagnosis | -0.1669 |
| Alcoholism | I(alc) | Takes value 1 if patient had a diagnosis of alcoholism in the past 365 days; takes value 0 if no diagnosis | 0.6985 |
| Anxiety | I(anx) | Takes value 1 if patient had a diagnosis of anxiety in the past 365 days; takes value 0 if no diagnosis | 0.1514 |
| Diabetes Mellitus Type 2 | I(dm2) | Takes value 1 if patient had a diagnosis of diabetes mellitus type 2 in the past 365 days; takes value 0 if no diagnosis | 0.2310 |
| Smoking | I(smo) | Takes value 1 if patient smoked in the past 365 days; takes value 0 if no smoking | 0.1932 |
| Urinary tract infectious disease | I(uri) | Takes value 1 if patient had a diagnosis of urinary tract ingectious disease in the past 365 days; takes value 0 if no diagnosis | 0.2436 |

To apply the OPEHR phenotype model to a person we must construct a linear function and compute its logit as follows:

Formula:

$$Y = -2.8289 + -2.3499\times I(a1) + -1.8485\times I(a2) + -1.2727\times I(a3) + -0.6051\times I(a4) + 0\times I(a5) +0.8051\times I(a6) + -0.0406\times I(male) + 0.1630\times I(ace) + 0.9237\times I(sei) + 0.1856\times I(ane) +0.2690\times I(epi) + -0.2363\times I(can) + 0.4278\times I(dep) + 0.0766\times I(copd) + 0.0909\times I(hf) + 0.1216\times I(kid) + -0.0464\times I(hyl) + -0.1381\times I(hyp) + -0.1669\times I(obe) + 0.6985\times I(alc) + 0.1514\times I(anx) + 0.2310\times I(dm2) + 0.1932\times I(smo) + 0.2436\times I(uri)$$

Then predicted 5-year risk P as a percentage is:

$$P = \frac{e^{Y}}{1+e^{Y}}\times100$$

The final model for the OMOP CDM is available in the *model/* directory at <https://github.com/mi-erasmusmc/DementiaPredictionModel>.
